# Yiqi Gubiao Pill for tuberculosis-associated obstructive pulmonary disease: protocol for a double-blind randomized controlled trial

**DOI:** 10.3389/fphar.2025.1610889

**Published:** 2025-10-08

**Authors:** Jin Dai, Yifan Zhang, Jun Su, Guoxiang Yuan, Fengsen Li, Zhian Zhou, Aziza, Chunying Ye, Aznamu Seyiti, Maihesebu Pida, Jian Zhang

**Affiliations:** ^1^ Karamay Integrated Traditional Chinese and Western Medicine Hospital (People’s Hospital of Karamay), Xinjiang, China; ^2^ School of Traditional Chinese Medicine, Beijing University of Chinese Medicine, Beijing, China; ^3^ Xinjiang Uygur Autonomous Region Hospital of Traditional Chinese Medicine, Xinjiang, China; ^4^ The Fourth Clinical Medical College of Xinjiang Medical University, Xinjiang, China; ^5^ Xinjiang Uygur Autonomous Region Research Institute of Traditional Chinese Medicine, Xinjiang, China; ^6^ Xinjiang Respiratory Disease Research Laboratory, Xinjiang, China; ^7^ Department of Infectious Diseases, People’s Hospital of Yecheng County, Xinjiang, China; ^8^ The Second People’s Hospital of Kuqa City, Xinjiang, China; ^9^ Department of Infectious Diseases, Shule County People’s Hospital, Xinjiang, China; ^10^ Department of Infectious Diseases, Shache County People’s Hospital, Xinjiang, China

**Keywords:** Yiqi Gubiao pill, active pulmonary tuberculosis, tuberculosis-associated obstructive pulmonary disease, double-blind randomized controlled trial, traditional Chinese medicine

## Abstract

**Background:**

Tuberculosis-associated obstructive pulmonary disease (TOPD), recognized as a high-morbidity respiratory condition in most countries, presents significant clinical challenges in differential diagnosis and comorbidity management, while substantially elevating all-cause mortality risk. Preclinical investigations of Yiqi Gubiao Pill (National Patent ZL201410536529.5) have demonstrated multi-target therapeutic efficacy, including cough suppression, bronchospasm alleviation, sputum expectoration facilitation, and disease progression retardation. This randomized controlled trial aims to systematically assess Yiqi Gubiao’s therapeutic and evaluate its safety.

**Methods:**

We will implement a prospective double-blind randomized controlled trial utilizing 1:1 allocation ratio, randomly assigned to either the Yiqi Gubiao pill treatment group or placebo-controlled group. Following randomization, a standardized 12-week therapeutic protocol will be administered, during which serial pulmonary function assessments and quality of life evaluations will be conducted. Concurrently, validated Traditional Chinese Medicine (TCM) symptom scoring scales will be applied for score. Systematic safety surveillance will be performed through weekly monitoring of adverse events.

**Discussion:**

This prospective, double-blind, randomized clinical trial will provide valuable data on the efficacy and safety of Yiqi Gubiao pill in treating TOPD. Positive results will offer a new treatment option for patients with TOPD.

**Clinical Trial Registration:**

[ClinicalTrials.gov], identifier [NCT06676800]. Registered 30 October 2024, https://ClinicalTrials.gov.

## Graphical Abstract

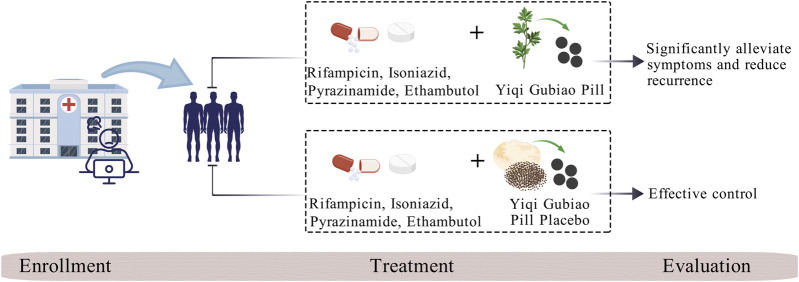

## 1 Introduction

According to the latest World Health Organization (WHO) report, tuberculosis (TB) has surpassed HIV/AIDS as the leading global cause of death from infectious diseases. In 2021 alone, an estimated 10.6 million people worldwide contracted TB, resulting in 1.6 million fatalities (Bagcchi, 2023). Notably, pulmonary tuberculosis (PTB) constitutes 80%–90% of all active TB cases. Existing research confirms that *Mycobacterium tuberculosis* has infected one-third of the global population, with approximately 10% of these latent infections progressing to active disease during the infected individuals’ lifetime (Dheda et al., 2016).

As one of the 30 high-burden TB countries designated by WHO, China contributes 9% of global tuberculosis cases, ranking second worldwide and surpassed only by India (Wang et al., 2012). Epidemiological monitoring reveals Xinjiang as the region with the most severe PTB burden in China, demonstrating an incidence rate 2.58 times higher than the national average (He et al., 2018). This disparity stems from multiple systemic challenges in the region, including geographical constraints, socioeconomic disparities, inadequate healthcare infrastructure, insufficient public health education, and critical shortages in TB prevention specialists (Qi, 2016). These compounding factors have driven a persistent escalation of TB transmission, establishing it as the most pressing public health challenge in northwestern China.

Recent clinical observations have identified a significant comorbidity between PTB and chronic obstructive pulmonary disease (COPD). Patients manifesting this specific comorbidity are clinically classified as having Tuberculosis-Associated Obstructive Pulmonary Disease (TOPD), a distinct diagnostic entity recognized in respiratory medicine (Sarkar et al., 2017). Comparative cohort studies demonstrate that TOPD patients exhibit distinct clinical trajectories compared to non-PTB-associated COPD cases: earlier disease onset (mean age reduction of 7.2 years), accelerated mortality timelines (hazard ratio 1.89), elevated exacerbation frequency (2.3-fold increase in annual hospitalization rates), and significantly diminished 10-year survival probabilities (34% vs 61%) (Yakar et al., 2017).

Current international TB management guidelines (Nahid et al., 2016) endorse the first-line therapeutic regimen comprising rifampicin, isoniazid, pyrazinamide, and ethambutol (RIPE protocol), supplemented by oxygen therapy in cases of concurrent obstructive ventilatory impairment. However, extended administration of first-line anti-TB agents precipitates dual therapeutic challenges: cumulative drug resistance development and suboptimal COPD comorbidity control. Therapeutic escalation to second-line agents (fluoroquinolones, diarylquinolines, and nitroimidazole derivatives including moxifloxacin, bedaquiline, and pretomanid) becomes imperative for multidrug-resistant TB. Nevertheless, pharmacovigilance data reveal significant neurotoxicity and idiosyncratic hepatotoxicity associated with prolonged use (Johnson et al., 2024). This therapeutic dichotomy has revitalized interest in TCM as adjuvant therapy, with meta-analytical evidence demonstrating adjunctive TCM protocols significantly improve clinical outcomes through three principal mechanisms: (1) reduction of standard chemotherapy cycle (2) accelerated sputum culture conversion rates and (3) decreased recurrence risk. Furthermore, longitudinal studies reveal TCM’s immunomodulatory properties mediate sustained pulmonary rehabilitation, such as enhance patient immunity and improve lung function (Luo and Cai, 2019; Huang and Hu, 2017).

Yiqi Gubiao Pill, a hospital-developed botanical formulation (Preparation Approval No. ZJ20130098; National Invention Patent License ZL201410536529.5; NMPA Clinical Trial Authorization 2018L02421), demonstrates multimodal therapeutic properties in preclinical studies. Pharmacodynamic evaluations reveal its significant antitussive, antiasthmatic, mucoactive, and immunopotentiating effects, with mechanistic studies indicating Yiqi Gubiao Pill’s capacity to downregulate serum concentrations of pro-inflammatory cytokines (Li et al., 2016). Bronchodilatory effects are mediated through reduction in airway resistance (Raw) and upregulation of pulmonary aquaporin-1 (AQP1) expression, elucidating its molecular mechanism in respiratory pathophysiology. This clinical trial (ClinicalTrials.gov ID: NCT06676800) implements a prospective double-blind, placebo-controlled randomized clinical trial (RCT) design to evaluate both therapeutic efficacy and safety profile in treatment-naive adults with active PTB complicated by COPD. This evidence-based approach aims to establish optimized integrative management protocols for this high-risk patient cohort. Meanwhile, through this three-phase model of “local problem-driven, scientific method validation, global demand adaptation,” Xinjiang’s TB research outcomes can serve as a pivotal nexus connecting China’s expertise with global tuberculosis control initiatives.

## 2 Methods and analysis

### 2.1 Study design

**FIGURE 1:**
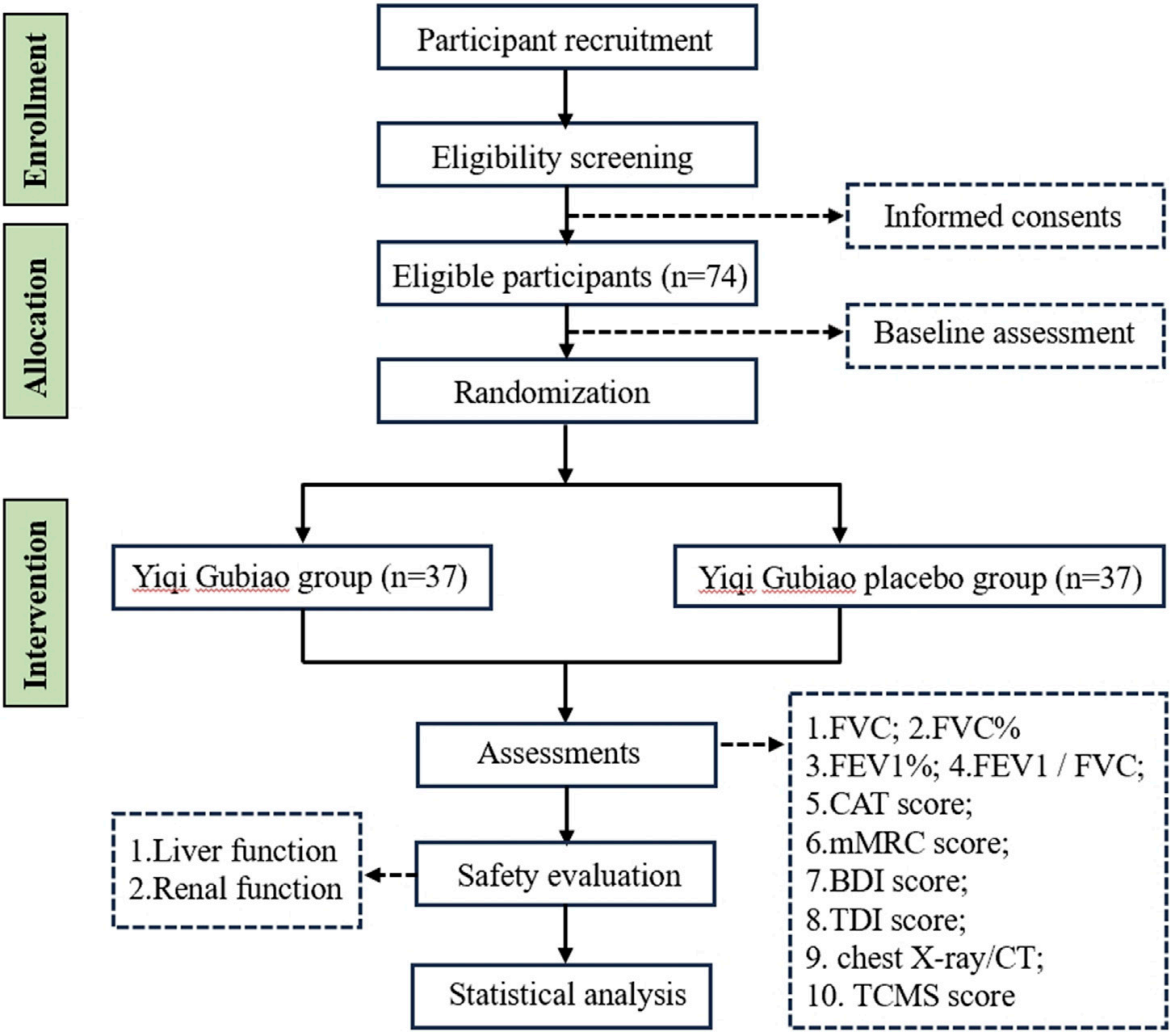
The flow chart of the experiment.

This prospective double-blind randomized controlled trial, registered with the Clinical Trials (registration number: NCT06676800), will implement a 1:1 allocation design randomized to either the Yiqi Gubiao treatment group or placebo-controlled group. Following randomization, a 12-week therapeutic protocol will be administered, during which serial pulmonary function assessments and quality of life evaluations. TCM syndrome differentiation will be quantified through the validated TCM Symptom Score Scale, while safety surveillance will systematically monitor adverse events. The trial adheres to SPIRIT guidelines (Standard Protocol Items: Recommendations for Interventional Trials), with the completed checklist provided in Supplementary Material 1. The complete study workflow is detailed in Figure 1.

### 2.2 Study setting

Study participants meeting the diagnostic criteria for active PTB with comorbid COPD will be recruited from respiratory medicine outpatient clinics and inpatient wards at the Sixth People’s Hospital of Xinjiang Uygur Autonomous Region. Diagnostic confirmation of active PTB will adhere to the Chinese National Health Commission’s Health Industry Standard WS 288-2017, requiring: 1) radiological evidence of active TB lesions on chest CT, and 2) microbiological confirmation through acid-fast bacilli (AFB) smear positivity with subsequent *Mycobacterium tuberculosis* growth on culture medium. All enrolled subjects will provide written informed consent following approval by the Sixth People’s Hospital of Xinjiang Uygur Autonomous Region Medical Ethics Committee (Approval No. 2021XE0138).

### 2.3 Participants

#### 2.3.1 Inclusion criteria

Patients who meet the following criteria will be included:

1. Aged 40-75 years old;
2. Meeting the Western medical diagnostic criteria and typing standards for active PTB;
3. No use of steroids, anti-tuberculosis medications, or immunosuppressants, nor desensitization therapy administered within the past month;
4. Documented comprehension of therapeutic protocols with voluntarily signed informed consent.

#### 2.3.2 Exclusion criteria

Patients who meet any of the following criteria will be excluded:

1. Patients with pneumothorax, pleural effusion, lung cancer, or other severe pulmonary comorbidities;
2. Patients with severe cardiovascular/cerebrovascular, hepatorenal, or hematopoietic system disorders;
3. Patients with psychiatric disorders;
4. Patients with neoplastic diseases;
5. Patients with congenital or acquired immunodeficiency.

#### 2.3.3 Rejection criteria

1. Patients with documented non-adherence to prescribed medication regimens, precluding reliable therapeutic effect assessment;
2. Patients withdrawing from the trial during the study period through self-initiated or legal guardian-requested discontinuation;
3. Patients using concomitant medications outside the approved trial protocol, particularly agents known to compromise trial integrity or confound efficacy assessments.

#### 2.3.4 Corresponding treatment for dropout and loss to follow-up cases

1. Upon occurrence of serious adverse events requiring physician-directed trial discontinuation, such cases shall be classified as treatment failures;
2. Disease progression during the trial period or emergence of confounding symptoms compromising outcome assessments, when leading to investigator-initiated termination, shall be categorized as therapeutic non-responders;
3. Protocol deviations exceeding acceptable thresholds (e.g., medication adherence poor) precluding reliable efficacy evaluation shall be excluded;
4. Cases where unblinding or emergency unblinding occurs during the trial.

### 2.4 Sample size estimation, blind and randomization

Sample size determination was conducted using methodology derived from prior RCT, indicating a minimum requirement of 31 participants per group. This calculation yields a baseline enrollment target of 62 subjects. To account for potential attrition (anticipated dropout rate = 20%) and ensure adequate power for intent-to-treat analysis, the final adjusted sample size was established at 74 participants.

This randomized controlled trial will enroll 74 participants allocated through SAS-generated block randomization with 1:1 assignment to either the intervention or control arm. Opaque sequentially numbered envelopes containing allocation codes will be managed by a head nurse. The head nurse on duty distributes the envelopes to the attending physicians according to the order of subjects’ enrollment, and the attending physicians assign the treatment plans to the patients according to the group numbers. The head nurse on duty assigns a sequence number based on the attending physician’s diagnosis. A designated person from the project team is responsible for distributing the treatment medication to the subjects. A specific person within the research team enters the data, and a teacher from the statistical consulting room is responsible for data analysis. When the results are ready, a third party is present to jointly unblind the results. In accordance with the principles of blinding, neither the patients, the attending physicians, nor the data statisticians and analysts are aware of the specific group assignments.

### 2.5 Intervention

#### 2.5.1 Composition, preparation, and administration of the trial medication

**FIGURE 2:**
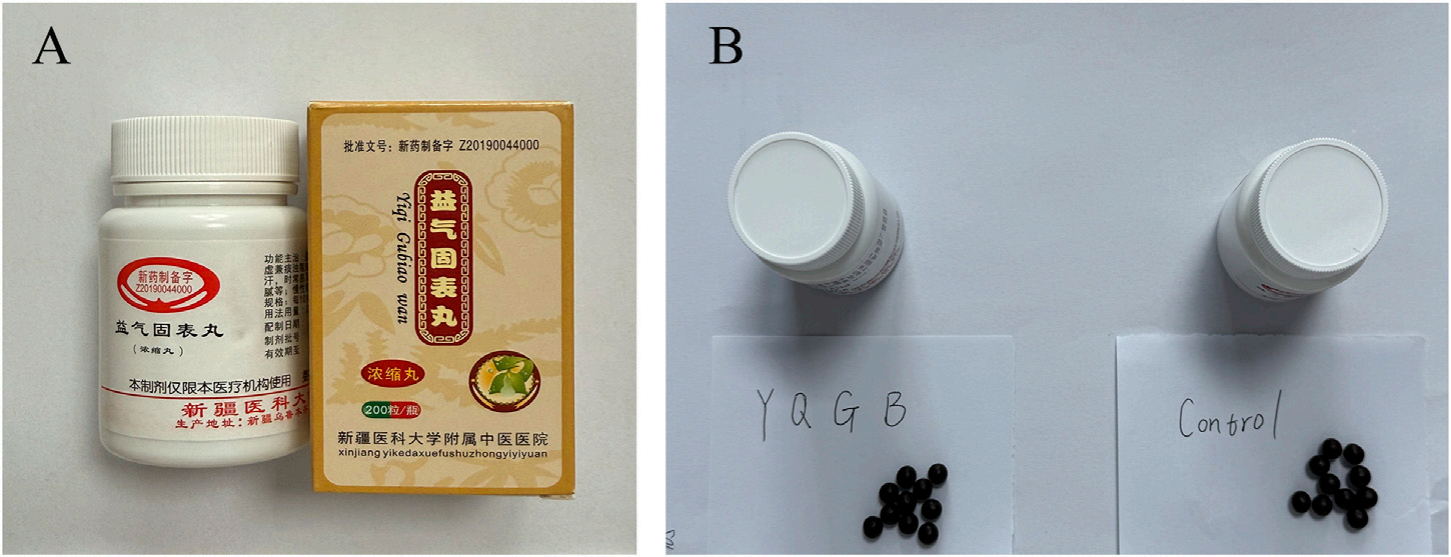
Yiqi Gubiao Pill and Control drug. **(A)** Appearance and Packaging of Yiqi Gubiao Pill; **(B)** Comparison of Yiqi Gubiao Pill and Placebo in Appearance.

The treatment group received Yiqi Gubiao Pill (specific composition detailed in Table 1; each pill containing 0.19 g, prepared by the Department of TCM Preparation, Affiliated Hospital of Traditional Chinese Medicine, Xinjiang Medical University). Administration consisted of 10 pills orally three times daily after meals, continued for 12 weeks. The control group received a placebo matching Yiqi Gubiao Pill in appearance and core color (primary ingredients: starch and caramel; prepared by the TCM Preparation Room, Affiliated Traditional Chinese Medicine Hospital of Xinjiang Medical University). Participants were instructed to take 10 placebo pills orally three times daily after meals for 12 weeks (Figure 2).

**TABLE 1:** The composition of Yiqi Gubiao Pill.

Chinese name	Latin name	Medicinal site
Dang-shen	Codonopsis radix	Root
Fu-xiao-mai	*Triticum aestivum*	Fruit
Bai-zhu	Atractylodes macrocephala Koidz	Rhizome
Ban-xia	Pinelliae rhizoma	Tuber
Chen-pi	Citri reticulatae pericarpium	Pericarp
Zi-su-zi	Perillae fructus	Fruit
Fu-ling	Poria cocos	Sclerotium
Yi-yi-ren	Coicis semen	Fruit
Fang-feng	Saposhnikoviae radix	Root
Kuan-dong-hua	Farfarae flos	Flower bud
Bei-mu	Fritillariae pallidiflorae bulbus	Bulblet
Huang-qin	Scutellariae radix	Root
Pi-pa-ye	Eriobotryae folium	Leafage

#### 2.5.2 Conventional treatment

Both treatment and control groups receive standardized anti-tuberculosis therapy following the WHO-recommended RIPE regimen—an intensive 8-week phase with daily rifampicin, isoniazid, pyrazinamide, and ethambutol, followed by a 16-week continuation phase with rifampicin and isoniazid—and concurrently receive guideline-concordant COPD management, including maintenance bronchodilator therapy.

### 2.6 Outcome

#### 2.6.1 Primary outcome

Lung function, long-term lung function decline and quality of life were established as primary efficacy endpoints.

##### 2.6.1.1 Lung function assessment​​

Pulmonary function testing (PFT) will be conducted under standardized protocols to ensure data validity and reproducibility. Patients will be instructed to abstain from inhaled bronchodilators for at least 24 h prior to testing to avoid transient airway dilation effects. Resting heart rate will be maintained below 120 bpm to minimize cardiovascular interference during spirometry. Key spirometric parameters included:

① Forced Vital Capacity (FVC)​​: Measures the total volume of air exhaled forcefully after maximal inhalation. Reduced FVC indicates severe obstructive conditions.
② FVC%​​: The ratio of measured FVC to predicted values (based on age, sex, and height). Values < 80% suggest significant impairment.
③ FEV1%​​: The percentage of predicted forced expiratory volume in the first second, reflecting airflow limitation severity. Classified as mild (≥80%), moderate (50%–79%), or severe (<50%).
④ FEV1/FVC Ratio​​: Critical for distinguishing obstructive (ratio <0.7) and restrictive (ratio ≥0.7 with reduced FVC) disorders.

##### 2.6.1.2 Long-term lung function decline

Long-term decline in lung function will be evaluated through serial spirometry conducted at baseline and predefined follow-up intervals (every 3 months). Patients with at least three valid pulmonary function tests over a 12- month period will be included in the analysis of decline trajectory. The following parameters will be used to quantify and classify lung function deterioration:

① Annual FEV_1_ Decline (mL/year): The change in forced expiratory volume in 1 s (FEV_1_) over time will be calculated and expressed per year. A decline greater than 60 mL/year will be defined as “rapid decline,” in alignment with thresholds reported in GOLD and SPIROMICS cohorts. Declines between 30 and 60 mL/year will be considered moderate, while declines under 30 mL/year will reflect physiological aging.
② Annual Change in FEV_1_% Predicted: The annual reduction in FEV_1_ as a percentage of predicted values will be computed. A reduction exceeding 5% per year will indicate clinically significant progression.
③ FVC and FVC% Predicted Trends: Longitudinal changes in forced vital capacity (FVC) and its percentage of predicted will be monitored to detect potential restrictive patterns or lung volume loss.
④ FEV_1_/FVC Ratio Decline: The ratio of FEV_1_ to FVC will be tracked over time. A progressive decrease in this ratio will reflect worsening airflow obstruction, particularly relevant in COPD progression.

##### 2.6.1.3 ​​Quality of life (QL) evaluation​​

QL was assessed using validated tools:

① COPD Assessment Test (CAT)​​: An eight-item questionnaire scoring symptoms (cough, dyspnea) and functional limitations (0–40 scale). A 5-point change indicates clinically meaningful improvement.
② mMRC Dyspnea Scale​​: Grades breathlessness from 0 (no dyspnea) to 4 (dyspnea at rest). Scores ≥2 correlate with increased hospitalization risk.
③ Baseline Dyspnea Index (BDI)​​ and ​​Transition Dyspnea Index (TDI)​​: BDI assesses dyspnea impact on daily activities (e.g., task difficulty), while TDI measures changes post-treatment. A TDI score ≥1 signifies meaningful improvement.
​​④ Chest Radiography​​: Screened for structural abnormalities (e.g., hyperinflation, effusions) but limited in detecting early small airway disease.
​​⑤ High-Resolution CT (HRCT)​​: Quantified emphysema extent, airway wall thickness, and fibrosis patterns. Dynamic CT evaluated ventilation-perfusion mismatches in advanced cases.
⑥ Acute Exacerbation of COPD (AECOPD): Exacerbation Frequency Classification: Based on events in the previous 12 months. Infrequent exacerbators: ≤1 exacerbation/year without hospitalization; Frequent exacerbators: ≥2 exacerbations/year or ≥1 requiring hospitalization.

#### 2.6.2 Secondary outcomes

Secondary outcomes will include TCM syndrome differentiation scoring, implemented per the Guidelines for Clinical Research of New Chinese Medicines (Trial). Symptomatology is categorized as follows: Core symptoms: Graded 0 (absent), 2 (mild), 4 (moderate), 6 (severe); Ancillary symptoms: Graded 0 (absent), 1 (mild), 2 (moderate), 3 (severe); Non-scored elements: Tongue manifestation and pulse characteristics. The composite TCM syndrome score is calculated through summation of all graded symptom scores.

#### 2.6.3 Safety assessment and adverse events reporting

An independent Data Safety Monitoring Board (DSMB)—comprising a pulmonologist, a clinical pharmacologist, and a biostatistician with no study roles—will oversee participant safety. The DSMB will review aggregate safety data at predefined intervals and *ad hoc* as needed, operating under a written charter with prespecified review procedures, communication pathways, emergency unblinding procedures, and stopping rules.

Safety surveillance will involve serial biochemical monitoring during pre-treatment, on-treatment, and post-treatment phases to establish the safety profile of Yiqi Gubiao Pill. All adverse events occurring during the study period will be prospectively monitored and systematically documented. Participants will be instructed to self-report potential adverse events in real-time, and researchers will determine whether the event is related to the study medication. If the adverse event is related to the study formulation, it is then referred to as a side effect. If the side effects are mild and the patient agrees to continue the treatment, medication will be continued after the symptoms have subsided. In case of serious adverse events due to the trial medication, the researcher will report to the DSMB and research ethics committee within 24 h, ask the patient to discontinue the medication, and implement emergency safety measures.

### 2.7 Data collection and management

Data collection will be carried out before, during, and after treatment (Table 2). Data will be recorded on pre-designed case report forms (CRF) and transferred to an electronic format (RevMan 5.3) for storage and data backup. All patient identity information will be encoded.

**TABLE 2:** Participant enrollment process, interventions, and outcome measures.

Items	Baseline	Treatment phase (monitor monthly)			Follow-up phase (monitor monthly)		
		1 month	2 months	3 months	3 months	6 months	12 months
Enrolment							
Recruitment	●						
Eligibility Screening	●						
Signed written informed consent	●						
Allocation	●						
Intervention							
Yiqi Gubiao pill (Treatment group)		●	●	●	●	●	●
Yiqi Gubiao pill Placebo (Control Group)		●	●	●	●	●	●
Outcomes							
forced vital capacity (FVC)	●	●	●	●	●	●	●
the percentage of FVC predicted (FVC%)	●	●	●	●	●	●	●
the percentage of forced expiratory volume in one second predicted (FEV1%)	●	●	●	●	●	●	●
FEV1/FVC	●	●	●	●	●	●	●
CAT score	●	●	●	●	●	●	●
mMRC score	●	●	●	●	●	●	●
BDI score	●	●	●	●	●	●	●
TDI score	●	●	●	●	●	●	●
chest X-ray/CT	●	●	●	●	●	●	●
Traditional Chinese Medicine Symptom (TCMS) score	●	●	●	●	●	●	●
Security							
All adverse events (including symptoms, signs, etc.)		●	●	●	●	●	●
Liver function: alanine aminotransferase, aspartate aminotransferase, total bilirubin, albumin, globulin	●	●	●	●	●	●	●
Renal function: uric acid, urea nitrogen, serum creatinine, β2-microglobulin, urinary protein, carbon dioxide binding rate, cystatin C	●	●	●	●	●	●	●

### 2.8 Data analysis

Two researchers independently entered observational data to establish a standardized database, with subsequent statistical analyses performed using SPSS 25.0. Quantitative variables exhibiting normal or approximately normal distributions are expressed as mean ± standard deviation (x̄±s). Between-group comparisons of means were analyzed via independent t-tests, while within-group longitudinal changes were assessed using paired t-tests. Categorical data were evaluated through χ^2^ tests or Wilcoxon rank-sum tests, and ordinal data were analyzed using non-parametric methods (e.g., Kruskal-Wallis test for multi-group comparisons). All statistical inferences employed two-tailed tests with a predefined significance threshold of α = 0.05.

### 2.9 Quality monitoring

Prior to study commencement, principal investigators delivered a standardized training program encompassing protocol-specified diagnostic criteria and biospecimen collection procedures outlined in the study protocol. Research personnel demonstrating competency through post-training assessments were granted authorization to execute protocol-specified procedures within predefined operational parameters.

## 3 Discussion

Tuberculosis remains a critical global public health threat, with its disease burden and therapeutic complexity exhibiting amplified effects in specific regions. Approximately one-third of the global population harbors latent Myc infection, forming a vast reservoir for potential active disease, while the emergence of comorbidities further complicates clinical management. In Northwest China, for instance, disparities in geographic conditions and healthcare resource allocation have fostered a region-specific TB epidemic pattern. The synergistic interaction between TB and chronic airway diseases accelerates pulmonary function decline and drastically diminishes quality of life. Although current international standardized regimens effectively suppress pathogen proliferation, prolonged drug exposure risks driving antimicrobial resistance, and inadequate control of comorbidities creates dual therapeutic barriers. Against this backdrop, traditional medicine offers novel perspectives through immunomodulatory mechanisms and pathological microenvironment repair, which may enhance treatment tolerance, alleviate symptoms, and reduce relapse rates.

This randomized controlled trial represents a pivotal effort to address the dual burden of active PTB complicated by secondary COPD in Xinjiang—a region disproportionately affected by TOPD. By integrating standardized anti-TB chemotherapy with Yiqi Gubiao Pill, the study design reflects a holistic approach to managing this high-risk population. This strategy could reduce reliance on prolonged first-line anti-TB regimens, potentially delaying or avoiding the need for more toxic second-line drugs. If validated, these findings would provide critical insights for optimizing TOPD treatment strategies, particularly in resource-constrained settings with systemic healthcare challenges.
